# Therapeutic mechanisms of mesenchymal stem cells in acute respiratory distress syndrome reveal potentials for Covid-19 treatment

**DOI:** 10.1186/s12967-021-02862-x

**Published:** 2021-05-10

**Authors:** Wendi Wang, Wei Lei, Lina Jiang, Siqi Gao, Shijun Hu, Zi-Gang Zhao, Chun-Yu Niu, Zhen-Ao Zhao

**Affiliations:** 1 Institute of Microcirculation, Hebei North University, 11 Diamond South-road, Keji Building, Room 213, Zhangjiakou, 075000 Hebei China; 2 Department of Pathophysiology of Basic Medical College, Hebei North University, Zhangjiakou, 075000 Hebei China; 3 Hebei Key Laboratory of Critical Disease Mechanism and Intervention, Shijiazhuang, 050017 Hebei China; 4 Hebei Key Laboratory of Critical Disease Mechanism and Intervention, Zhangjiakou, 075000 Hebei China; 5 Pathophysiology Experimental Teaching Center of Basic Medical College, Hebei North University, Zhangjiakou, 075000 Hebei China; 6 Department of Cardiovascular Surgery of the First Affiliated Hospital & Institute for Cardiovascular Science, State Key Laboratory of Radiation Medicine and Protection, Medical College, Soochow University, Suzhou, 215000 Jiangsu China; 7 Basic Medical College, Hebei Medical University, Shijiazhuang, 050017 Hebei China

**Keywords:** Mesenchymal stem cells, Covid-19, Acute respiratory distress syndrome

## Abstract

The mortality rate of critically ill patients with acute respiratory distress syndrome (ARDS) is 30.9% to 46.1%. The emergence of the coronavirus disease 2019 (Covid-19) has become a global issue with raising dire concerns. Patients with severe Covid-19 may progress toward ARDS. Mesenchymal stem cells (MSCs) can be derived from bone marrow, umbilical cord, adipose tissue and so on. The easy accessibility and low immunogenicity enable MSCs for allogeneic administration, and thus they were widely used in animal and clinical studies. Accumulating evidence suggests that mesenchymal stem cell infusion can ameliorate ARDS. However, the underlying mechanisms of MSCs need to be discussed. Recent studies showed MSCs can modulate immune/inflammatory cells, attenuate endoplasmic reticulum stress, and inhibit pulmonary fibrosis. The paracrine cytokines and exosomes may account for these beneficial effects. In this review, we summarize the therapeutic mechanisms of MSCs in ARDS, analyzed the most recent animal experiments and Covid-19 clinical trial results, discussed the adverse effects and prospects in the recent studies, and highlight the potential roles of MSC therapy for Covid-19 patients with ARDS.

## Background

Acute respiratory distress syndrome (ARDS) remains the leading cause of mortality in critically ill patients. The in-hospital mortality rate for ARDS is 34.9%–46.1% [1]. According to the Berlin Definition, ARDS can be categorized into three degrees as mild, moderate, and severe based on the degree of hypoxemia [2]. The causes of ARDS include severe pneumonia, sepsis, trauma, hemorrhagic shock, reperfusion injury, influenza virus, coronavirus and so on [3–5].

ARDS is characterized by increased pulmonary capillary endothelial cell and alveolar epithelial cell permeability, inflammatory cell infiltration, lung edema, impairment of oxygenation and pulmonary fibrosis. The main treatments for ARDS patients include mechanical ventilation, using diuretic to reduce pulmonary edema, and prone positioning to improve pulmonary gas exchange [6, 7]. Despite the therapeutic progress, more effective approaches are urgently needed for ARDS.

MSCs are multipotent adult stem cells derived from various tissues and organs, including bone marrow (BM), adipose (AD) and umbilical cord (UC). MSC-based therapies are widely used for the treatment of several diseases in pre-clinical models and under investigation in many clinical trials [8–10]. After infusion via veins, MSCs show a tropism for lung tissue due to hemodynamic matter within 5 min. The cell retention time in the lungs ranged from hours to days in different studies [11–15]. Some studies demonstrated that these cells can stay even less than 24 h in the lungs, although still exerting their therapeutic actions [12]. Thus, MSCs have notable strengths for the treatment of lung diseases. Substantial preclinical studies have suggested that infusion of MSCs in animal models exhibits protective effects following ARDS, but the diversity of the mechanisms needs further discussion [16–18].

Furthermore, the global spreading coronavirus disease 2019 (Covid-19) is caused by severe acute respiratory syndrome coronavirus 2 (SARS-CoV-2), which is a type of RNA virus belonging to the coronaviridae family [19]. ARDS is the main cause of death in critically ill patients with Covid-19 [20]. MSC transfusion is anticipated to be a feasible therapy for severe or critically ill Covid-19 patients.

Here, we summarized the current understanding of therapeutic mechanisms of MSC-based treatments on ARDS. The progress and limitations of MSC therapy in the most recent pre-clinical research and clinical applications were discussed in this review. These results shed light on the treatment of Covid-19.

## Immunomodulatory properties of MSCs in ARDS

Neutrophils have been recognized as the drivers of pathophysiology in ARDS, releasing several pro-inflammatory mediators associated with direct injury to the lung tissue [21]. As neutrophils migrate across the epithelial cells, some toxic mediators are released by neutrophils such as proteases, neutrophil extracellular traps (NETs), and reactive oxygen species (ROS) [6]. Over-production of ROS by neutrophils is also called oxidative burst or respiratory burst [22], which not only kills pathogens but also harms pulmonary vascular endothelium and alveolar epithelium [6]. Mouse AD- and human BM-MSCs infusion can inhibit neutrophil activation and lead to a reduction of ROS [23, 24]. Furthermore, mouse AD-MSCs inhibit the release of NETs which are part of the neutrophil response, thus inhibiting nuclear factor kappa-B (NF-κB) and improving the survival rates in ARDS [24–26]. Interestingly, Human and mouse BM-MSC-conditioned medium also could induce neutrophil apoptosis via inhibiting the NF-κB signaling pathway to alleviate lung injury [27], indicating the paracrine function of MSCs may play important roles in lung repair.

Macrophages show a dynamic balance between M1-type (classically activated macrophage) and M2-type (alternatively activated macrophage) polarization during ARDS. The M1 subtype releases pro-inflammatory cytokines, including TNF-α, IFN-γ, IL-1β, IL-6, IL-12, and IL-23, and expresses inducible nitric oxide synthase (iNOS), contrarily, the M2 subtype secretes anti-inflammatory cytokines, including IL-4, IL-10 and TGF-β [28–31]. After noncontact coculture with human BM-MSCs, the macrophages showed increased M2 polarization and phagocytic capacity. This may explain the anti-inflammatory effects of human BM-MSCs in lipopolysaccharide (LPS)-induced mouse lung injury [32]. Furthermore, human AD-MSC-educated macrophages could increase the levels of IL-4 and IL-10, and reduce the levels of TNF-α and IL-6 in the serum and bronchoalveolar lavage fluid, thereby ameliorating the LPS-induced systemic inflammatory response in a mouse model [33]. Therefore, these results revealed human AD-MSCs exert anti-inflammatory roles through regulating M2 polarization. In addition, macrophages incubated with human BM-MSCs showed higher phagocytotic activity in *Escherichia coli*-induced lung injury in rats [34]. Interestingly, human BM-MSCs may transfer mitochondria to macrophages via tunneling nanotubes and extracellular vesicles to enhance macrophage oxidative phosphorylation, contributing to the antimicrobial effect and phagocytic activity of macrophages in ARDS [32, 35].

During ARDS that induced by hemorrhagic shock or LPS, the number of dendritic cells (DCs) in lung tissue is increased, and the maturation of pulmonary DCs participates in aggravating lung inflammatory response and pathological injury [36–38]. Hound AD- and mouse BM-MSCs could induce mature dendritic cells (mDCs) into regulatory dendritic cells (DCregs) population, leading to the suppression of mDCs activation and inhibition of inflammatory cytokines secretion in vitro [39, 40]. Mechanically, paracrine hepatocyte growth factor (HGF) secreted by mouse BM- and human UC-MSCs can activate the AKT signaling pathway, inducing mDCs differentiation into DCregs and inhibit T cell proliferation to ameliorate lung injury in murine models [41, 42].

T helper 17 (Th17) cells and regulatory T cells (Treg cells) also play roles in ARDS [43–45]. An increased ratio of Th17/Treg cells is correlated with poor prognosis in ARDS patients, and it is also a novel risk indicator to determine 28-day mortality [46]. Mouse BM-MSCs could regulate the polarization of T cells into Th17 and Treg, reduce the Th17/Treg ratio, and balance inflammatory cytokines in vivo and in vitro [47, 48]. Rat lung-resident MSCs can also attenuate lung injury through decrease Th17 cells and increase Treg cells in a mouse model. Correspondingly, MSCs decrease Th17-related cytokines IL-17 and IL-22, and increase Treg-related IL-10 expression in both lung and plasma [44] In vitro coculture study showed mouse BM-MSCs inhibited the differentiation of Th17 cells from naive CD4^+^ T cells via the programmed death-1 (PD-1) pathway through cell-to-cell contact [49]. However, this should be further verified in vivo using ARDS models.

**Fig. 1:**
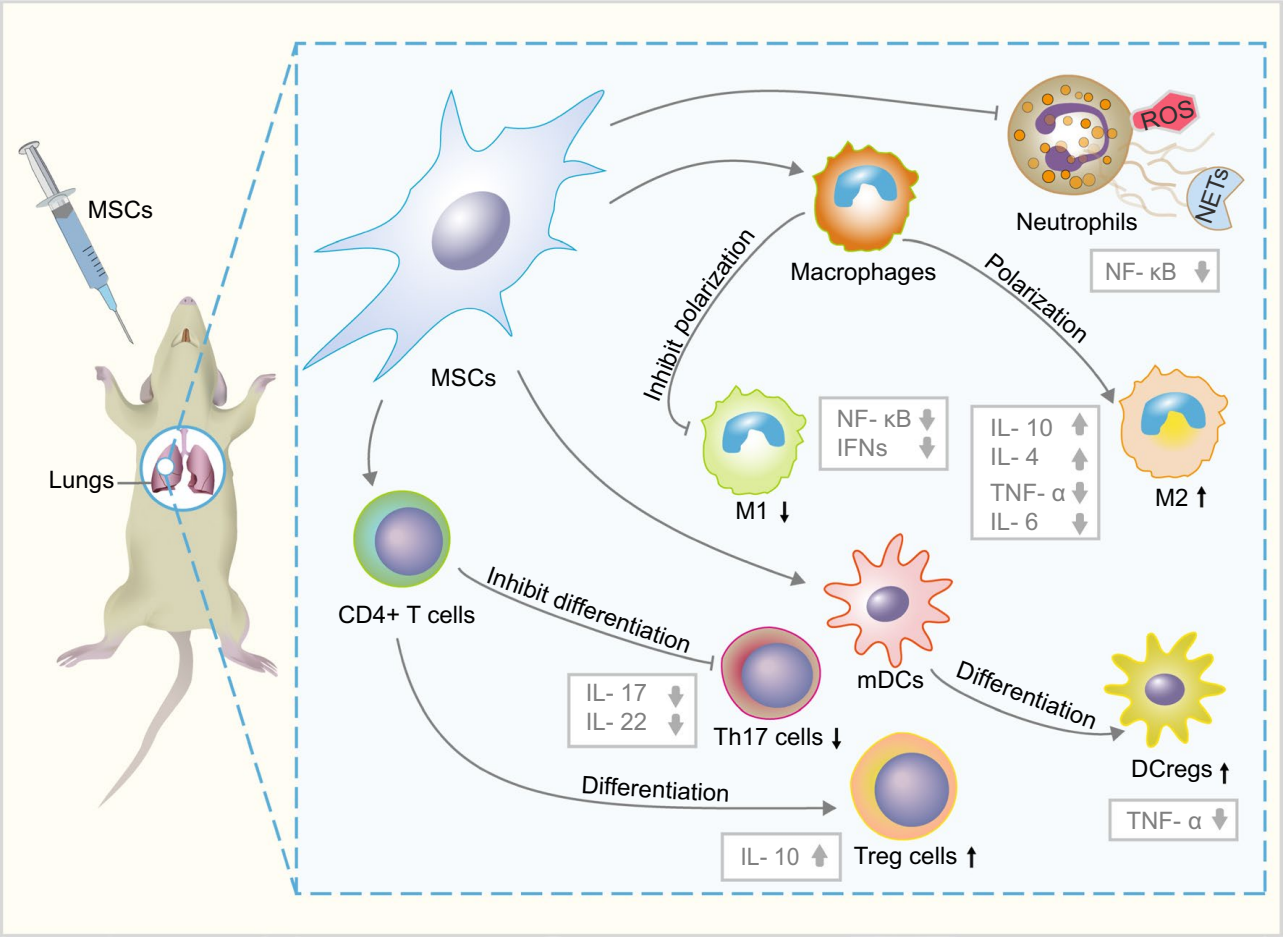
MSCs remedy ARDS through the regulation of immune and inflammatory cells. *DCregs* regulatory dendritic cells, *mDCs* mature dendritic cells, *ROS* reactive oxygen species, *NETs* neutrophil extracellular traps, *Th17* T helper 17, *Treg cells* regulatory T cells,* M1* M1 macrophage, * M2* M2 macrophage

Taken together, MSCs can reduce inflammatory tissue damage in ARDS by modulation of immune and inflammatory cells (Fig. 1). However, the molecular mechanism of MSCs in the inflammatory response is still unclear.

## Paracrine function of MSCs in maintaining the alveolar epithelial and endothelial barrier

Pulmonary vascular endothelium is a monolayer of endothelial cells arranged on the vessel luminal surface and is responsible for endothelial barrier function. Dysfunction of pulmonary vascular endothelial barrier is associated with increased endothelial permeability and lung edema. There are two main pathways to regulate the permeability across the vascular endothelial barrier: paracellular and transcellular [50]. Paracellular permeability is determined by the junction proteins, such as β-catenin, VE-cadherin, and occludin, while transcellular permeability is indirectly reflected by the endothelial barrier macromolecules, such as transferrin and albumin [51, 52].

In vitro experiments showed that the human BM-MSC-conditioned medium could restore pulmonary endothelial permeability by maintaining adherens junction proteins (VE-cadherin and β-catenin) [52], indicating paracrine factors in the conditioned medium could regulate pulmonary endothelial permeability. Recently, an in vitro study found that the pulmonary endothelial paracellular permeability was increased after stimulated by LPS, and was restored after noncontact coculture with mouse BM-MSCs. Mechanically, this study confirmed that mouse BM-MSCs secreted HGF as paracrine factor to protect tight junction protein occludin and endothelial barrier through mTOR/STAT3 signaling pathway [51]. Another similar study showed synergism of human MSC-secreted paracrine factors HGF and vascular endothelial growth factor (VEGF) protected paracellular and transcellular endothelial barrier by activating Rac1 signaling pathway [53].

Besides, paracrine factors secreted by MSCs can protect the alveolar epithelial integrity. In the injured alveoli, the epithelial barrier dysfunction leads to the protein-rich edema formation and accumulation of inflammatory cells, which results in a further decrease of Na^+^ absorption across the alveolar epithelium and more serious damage of type II alveolar epithelial cells (AEC II) [54].

In vitro study showed that human BM-MSC-conditioned medium reversed epithelial hyperpermeability and restored transepithelial Na^+^ transport. Additionally, the paracrine keratinocyte growth factor (KGF) secreted into the conditional medium from human BM-MSCs was required for the protective effect on alveolar epithelial Na^+^ transport [55]. Moreover, epithelial permeability was increased when AEC II was exposed to inflammatory insults (the combination of IL-1β, TNF-α and IFN-γ), while the paracrine factor angiopoietin-1 (ANG-1) secreted by the cocultured human BM-MSCs could restore epithelial integrity [56].

**Fig. 2:**
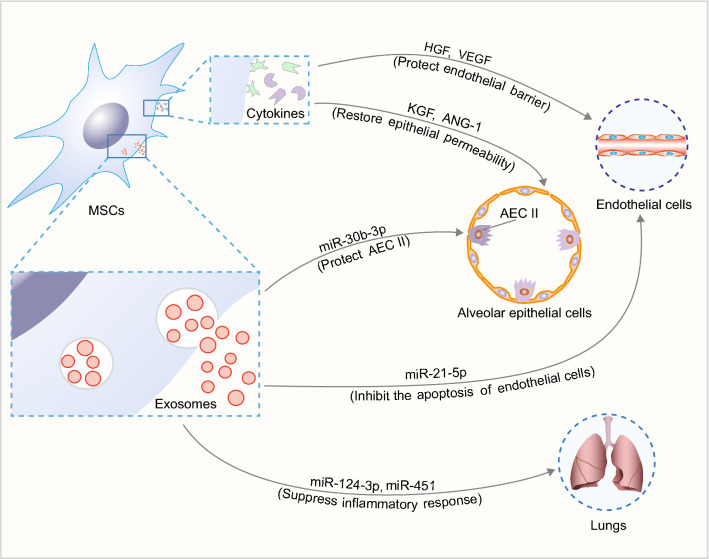
Effects of MSC-derived paracrine factors on ARDS. *HGF* hepatocyte growth factor, *VEGF* vascular endothelial growth factor, *KGF* keratinocyte growth factor, *ANG-1* angiopoietin-1, *AEC II* type II alveolar epithelial cells

These studies indicated that MSC-derived paracrine factors are effective stabilizers of pulmonary vascular endothelium and alveolar epithelium (Fig. 2). However, these mechanisms should be further verified in vivo.

## Therapeutic potential of MSC-derived exosomes in ARDS

Exosomes are nano-sized extracellular vesicles (30–100 nm in diameter) that are actively secreted by various cells including MSCs. They carry therapeutic cargos such as proteins, miRNAs and mRNAs, and can transfer these biological molecules to target cells to affect their biological properties [57]. The therapeutic benefits of MSC-exosomes have been shown in several aspects of ARDS (Fig. 2).

MSC-derived exosomes were demonstrated to mediate the inflammatory responses and regulate immune function in ARDS. P2X ligand-gated ion channel 7 (P2X7) is closely involved in the inflammatory process of ARDS. Rat BM-MSCs-derived exosomes carry miR-124-3p to inhibit P2X7 expression, suppress the inflammatory response, and ameliorate traumatic ARDS [58]. Rat BM-MSC-derived exosomes could also inhibit the TLR4/NF-κB signaling pathway, and suppress intestinal ischemia reperfusion-induced ARDS [59]. Consistently, exosomes from human UC-MSCs could transfer miR-451 to downregulate the expression of TLR4 and p65, and thus restricted the TLR4/NF-κB signaling pathway in burn-induced ARDS [60]. Furthermore, mouse BM-MSC-derived exosomes can inhibit pulmonary endothelial apoptosis through miR-21-5p, which targets PDCD4 and PTEN [61]. Besides, engineered exosomes represent a new direction. Mouse BM-MSC-derived exosomes overexpressing miR-30b-3p could relieve the inflammation reaction and repair AEC II by inhibiting serum amyloid A3 (SAA3), which has been considered as an inflammatory acute phase reactant [62]. Above all, MSC exosome-related miRNAs play important roles in ARDS, representing a promising non-cellular therapeutic strategy.

In addition, MSC-exosomes can regulate the metabolic state of alveolar macrophages. Mouse BM-MSC-derived exosomes could inhibit HIF-1α to downregulated the glycolysis, and thus inhibit M1 macrophage polarization and promote M2 macrophage polarization in lung tissue, which ameliorated the LPS-induced ARDS [63]. This mechanism might be synergetic with the role of BM-MSCs in macrophage oxidative phosphorylation through mitochondria transfer [32].

## Role of MSCs in endoplasmic reticulum stress

Numerous studies have found that inhibition of endoplasmic reticulum stress (ERS) could prevent or reduce ARDS [64–66]. Blocking ERS with 4-phenyl butyric acid can significantly ameliorate apoptosis and histopathological alterations in lung tissue [65]. Besides, inhibition of ERS also prevented the activation of NF-κB signaling pathway and decreased pro-inflammatory mediators, including TNF-α, IL-1β, and IL-6 [66].

In vitro study showed ERS induced by bleomycin can promote the AEC apoptosis, while mouse BM-MSC-conditioned medium could attenuate AEC injury by reducing ERS [67]. In in vivo studies, the levels of ERS markers (Bip or XBP-1) in AEC and fibroblast were elevated since day 7 after bleomycin-induced lung injury. Human BM-MSC infusion through vein could inhibit ERS mainly through Bip-PERK-Nrf2 pathway, while the other two sensors located in endoplasmic reticulum membrane were not affected by human BM-MSC infusion, including inositol-requiring enzyme 1 (IRE-1) and activating transcription factor 6 (ATF-6). Surprisingly, human BM-MSCs did not affect the ERS-induced apoptosis [68]. These studies showed inconsistent results between in vitro and in vivo experiments. We speculated the intravenous infusion route may affect the efficiency of MSC therapy for AEC, because the MSCs contact with lung endothelial cells firstly, and must penetrate endothelial cell barrier to reach AEC.

## Anti-fibrotic capacity of MSCs in ARDS

Pulmonary fibrosis is a progressive interstitial lung disease caused by many reasons, including viral and bacterial infections, adverse reactions of chemotherapy drugs, and environmental factors such as air pollution, smoking, and occupational exposures. Intra-alveolar and interstitial fibrosis are hallmarks in the late stage of ARDS which are manifested as the abnormal deposition of extracellular matrix proteins, especially collagen. Lung fibrosis mainly involves two cellular mechanisms. The inflammatory lung environment in ARDS may trigger epithelial-mesenchymal transition of AEC II, which differentiates into active myofibroblasts [69]. Besides, TGF-β-induced transformation of fibroblasts to myofibroblasts contributes to lung fibrosis [70]. Pulmonary fibrosis can severely affect the ARDS patients with accelerated lung dysfunction, leading to ventilator dependence [71]. Hence, decreasing fibrosis of the lung is imperative to prevent ARDS.

Intratracheal infusion of human AD-MSCs could significantly ameliorate lung injury by attenuated interstitial fibrosis in LPS-induced ARDS mouse models and reducing neutrophil infiltration [72]. Similarly, intravenous infusion of human UC-MSCs inhibited bleomycin-induced fibrosis in immunocompetent mice [70]. In mechanism, human UC-MSCs can reverse fibrosis through enhanced expression of macrophage matrix-metallopeptidase-9 for collagen degradation, and enhanced toll-like receptor-4 signaling pathway for alveolar regeneration [69]. Moreover, intravenous infusion of rat AD-MSCs reduced the expression of fibroblast growth factor-7 in serum and lung tissue, reversing the process of fibrosis in amiodarone-induced lung injury [73]. Additionally, mouse BM-MSCs with Last-1 or Last-2 knockdown exhibit a stronger antifibrotic ability at the early stage of LPS-induced ARDS [74, 75]. These studies demonstrated that MSCs from different tissues could remedy ARDS by attenuating pulmonary fibrosis.

## Clinical trials

Injection of MSCs is a promising therapy for the treatment of ARDS in pre-clinical models (Table 1), but MSC-based therapies still under investigation in clinical trials. Due to the progress in pre-clinical studies, several clinical trials were registered to investigate the safety and efficacy of allogeneic MSC therapy in ARDS patients, especially during the pandemic of Covid-19 (Table 2).

**Table 1:** Pre-clinical studies: MSCs transplantation in ARDS

Study	Injury model	Source	Dosage	Route of delivery	Primary outcomes
Florian et al. (2021)	Mouse-ARDS/LPS	Mouse BM-MSCs	2.5 × 10^5^	I.V	↓Neutrophils in BALF (~ 28% decrease), lung inflammation (TNFα: ~ 43% decrease, IFNγ: ~ 73% decrease)
Zhang et al. (2020)	Mouse-ARDS/LPS	Mouse BM-MSCs	5 × 10^5^	I.V	↑ATP levels (~ 40% increase), oxygen consumption rate (~ 95% increase) ↓Lung inflammation (IL-1β: ~ 58% decrease, TNFα: ~ 29% decrease), reactive oxygen species
Yudhawati et al. (2020)	Mouse-ARDS/H5N1 virus	Mouse BM-MSCs	5.5 × 10^5^	I.V	↑PaO2/FiO2 ratio (~ 30% increase) ↓Protein in BALF (~ 58% decrease), lung inflammation (NFκB: ~ 47% decrease, TNFα: ~ 55% decrease)
Sadeghian et al. (2020)	Sheep-ARDS/LPS	Sheep BM-MSCs	5 × 10^7^	I.T	↑PO2 (~ 21% increase), SatO2 (~ 6% increase) ↓Neutrophils in BALF (~ 22% decrease), PCO2 (~ 14% decrease)
Lu et al. (2020)	Mouse-ARDS/LPS	Mouse BM-MSCs	5 × 10^5^	I.V	↑DCregs ↓mDCs
Cheng et al. (2020)	Rat-ARDS/LPS	Rat LR-MSCs	2 × 10^6^	I.T	↓Lung inflammation (TNFα: ~ 36% decrease, MCP-1: ~ 31% decrease, IL10: ~ 32% increase)
Chen et al. (2020)	Mouse-ARDS/LPS	Mouse BM-MSCs	2 × 10^5^	I.T	↓Lung inflammation (IL-17A: ~ 33% decrease, IL10: ~ 25% increase), Th17/Treg (~ 53% decrease)
Radwan et al. (2020)	Rat-lung injury/Amiodarone	Rat AD-MSCs	2 × 10^6^, 4 × 10^6^	I.V	↓Pulmonary fibrosis
Jung et al. (2019)	Mouse-ARDS/LPS	Human AD-MSCs	2 × 10^5^	I.V	↓Lung injury score (~ 44% decrease)
Dong et al. (2019)	Mouse-ARDS/LPS	Mouse BM-MSCs	5 × 10^4^	I.T	↓Lung edema (~ 27% decrease), pulmonary fibrosis

*AD-MSCs* adipose-derived mesenchymal stem cells, *AECs* alveolar epithelial cells, *ARDS* acute respiratory distress syndrome, *BALF* bronchial alveolar lavage fluid, *BM-MSCs* bone marrow-derived mesenchymal stem cells, *DCs* dendritic cells, *IFNs* interferons, *I.P. *intraperitoneal, *I.T. *intratracheal, *I.V. *Intravenous, *LR-MSCs* lung-resident mesenchymal stem cells, *LPS* lipopolysaccharides, *NETs* neutrophil extracellular traps, *mDCs* mature dendritic cells, *DCregs* regulatory dendritic cells, *Th17* T helper 17, *Tregs* regulatory T cells, *UC-MSCs* umbilical cord-derived mesenchymal stem cells

**Table 2:** Clinical trials: Registered MSC-based treatment in ARDS

Identifier (status)	Disease	Phase	Cell source	Dosage	Route	Enrolled number	Primary outcomes
NCT01775774 (Completed)	ARDS	1	BM-MSCs	1, 5, 10 × 10^6^ cells/kg	I.V	9	Infusion associated adverse events
NCT02097641 (Completed)	ARDS	2a	BM-MSCs	1 × 10^7^ cells/kg	I.V	60	Infusion associated adverse events, numbers of death within 24 h
NCT01902082 (Unknown)	ARDS	1	AD-MSCs	1 × 10^6^ cells/kg	I.V	20	Adverse events
NCT02804945 (Completed)	ARDS in patients with malignancies	1	BM-MSCs	3 × 10^6^ cells/kg	I.V	20	Adverse events
ChiCTR2000029990 (Recruiting)	Covid-19-related pneumonitis	1–2	BM-MSCs	1 × 10^6^ cells/kg	I.V	60	Blood oxygen saturation
NCT04355728 (Recruiting)	Covid-19 patients	1–2	UC-MSCs	1 × 10^8^ cells (2 times)	I.V	24	Adverse events
NCT03042143 (Recruiting)	Covid-19-related ARDS	1–2	UC-MSCs	1, 2, 4 × 10^8^ cells	I.V	75	Oxygenation index, adverse events
NCT04390139 (Recruiting)	Covid-19-related respiratory distress	1–2	WJ-MSCs	1 × 10^6^ cells/kg	I.V	30	All-cause mortality at day 28
NCT02095444 (Recruiting)	H7N9-related ARDS	1–2	MB-MSCs	1 × 10^7^ cells/kg (4 times)	I.V	20	The degree of lung injury within 14 days
NCT04416139 (Recruiting)	Covid-19-related ARDS	2	UC-MSCs	1 × 10^6^ cells/kg	I.V	10	PaO2/FiO2 ratio, heart rate, respiratory rate, changes in body temperature
NCT04366063 (Recruiting)	Covid-19-related ARDS	2–3	BM-MSCs	1 × 10^8^ cells (2 times)	I.V	60	Adverse events, blood oxygen saturation
NCT04371393 (Recruiting)	Covid-19-related ARDS	3	BM-MSCs	2 × 10^6^ cells/kg (2 times)	I.V	300	All-cause mortality at day 30

*AD-MSCs* adipose-derived mesenchymal stem cells, *BM-MSCs* bone marrow-derived mesenchymal stem cells, *I.V. *intravenous, *MB-MSCs* menstrual blood-derived mesenchymal stem cells, *UC-MSCs* umbilical cord-derived mesenchymal stem cells, *WJ-MSCs* Wharton-Jelly mesenchymal stromal cells

In 2013, a phase I, multi-center and open-label clinical trial (NCT01775774) was started to test the safety of human BM-MSCs in ARDS patients. Nine patients received a single dose intravenous infusion of either 1, 5 or 10 million cells/kg predicted body weight (PBW). This trial confirmed the safety of BM-MSCs in the ARDS patients, with no BM-MSC-related adverse events occurring after infusion [76]. Subsequently, this team performed a multi-center and double-blind phase II clinical trial (NCT02097641) to evaluate the safety of the human BM-MSC-based therapy. Sixty participants were randomly assigned with a 2:1 ratio to receive either allogeneic BM-MSCs or placebo. BM-MSCs were administered intravenously at a dose of 10 million cells/kg PBW. There were no BM-MSC-related adverse events, however, efficacy should be further verified in larger trials. Meanwhile, the viability of BM-MSCs must be improved [77].

In 2015, BM-MSCs were used in a phase I open-label clinical trial for patients with septic shock, which is often related with ARDS (NCT02421484). Nine participants were randomly divided into three groups to receive a single intravenous BM-MSC infusion of 0.3, 1 or 3 million cells/kg PBW. The infusion of BM-MSCs into participants with septic shock appears safe and shows potential signs of efficacy [78].

Recently, Chen reported a single-center and open-label clinical study (NCT02095444) and evaluated allogeneic menstrual blood-derived MSC administration in patients with H7N9-induced ARDS. In this trial, 9 patients received 3 infusions of human menstrual blood-derived MSCs and 8 patients received 4 infusions of the cells. Menstrual blood-derived MSCs were intravenously injected at a dose of 1 million cells/kg PBW each time. The results showed that mortality was significantly lower in the MSC group (17.6% in MSC group *vs* 54.5% in control group). Furthermore, the 5-year follow-up survey in 4 patients showed the injection of menstrual blood-derived MSC was safe [79]. Therefore, the efficacy of MSC injection in H7N9-induced ARDS indicated the therapeutic potential of MSCs in Covid-19 patients.

Zheng and colleagues reported a phase I, single-center and double-blind study (NCT01902082). In this trial, 12 adult ARDS patients were randomly divided at a 1:1 ratio to receive either allogeneic human AD-MSCs or placebo. AD-MSCs were intravenously administrated at a dose of 1 million cells/kg PBW. However, the administration of AD-MSCs did not significantly improve pulmonary function. Meanwhile, the levels of serum inflammatory cytokines (IL-6 and IL-8) were not affected [80]. In fact, most of the existing related clinical trials were only in Phase I or Phase II and designed to evaluate safety as primary outcomes. Therefore, this may be underpowered for evaluating efficacy. Meanwhile, the efficacy of MSC-based therapy may be affected by various factors, including the sources of MSCs, cell viability, cell dosage, times of administration and delivery route [81, 82]. Therefore, the procedure for MSC production and transfusion should be standardized. Larger and well-controlled clinical trials are needed.

## The therapeutic potential of MSCs in Covid-19 patients

Severe pneumonia and ARDS have been observed in many Covid-19 patients. Among the affected patients who require hospitalization, the mortality may be in the range of 5%–15% [83]. However, these numbers are continually changing as the pandemic spread around the world. Proposed five key mechanisms are related to Covid-19 pathophysiology, including (1) the direct cytotoxicity of SARS-CoV-2 in epithelial cells; (2) dysregulation of the renin–angiotensin–aldosterone system caused by angiotensin-converting enzyme 2 (ACE2) downregulation resulted from the interaction of SARS-CoV-2 with ACE2; (3) dysregulation of immune response, and hyper-inflammation caused by cytokines and chemokines; (4) endothelial cell damage and thrombo-inflammation; (5) interstitial thickening and fibrosis [84, 85]. However, the detailed mechanisms in the pathophysiology of Covid-19 are still unclear currently.

Convalescent plasma holds great potential to treat Covid-19. Early high-titer plasma infusion could prevent severe Covid-19 in older adults [86]. A recent report showed the risk of death within 30 days was also associated with the anti-SARS-CoV-2 antibody levels in plasma transfusion. When patients were not receiving mechanical ventilation, the Covid-19 patients transfused with plasma with high-titer antibodies showed a lower risk of death than the low-titer group. However, among patients who were receiving mechanical ventilation, the risk of death was not associated with the antibody titer [87]. Disappointingly, four antiviral drugs including hydroxychloroquine, interferon beta-1a, lopinavir and remdesivir had little or no effect on hospitalized Covid-19 patients [88]. Therefore, effective therapy is urgently needed for Covid-19 patients with ARDS.

MSCs possess the ability for tissue regeneration and have the potential to suppress cytokine storm, pulmonary fibrosis in ARDS [69, 70, 89, 90], which were matched to fight against Covid-19. Thus, MSCs have drawn much attention for the treatment of Covid-19 patients. There is a rapidly growing number of clinical trials of MSC-based therapy approaches for Covid-19 (Table 2). A Phase I-II and multi-center study (ChiCTR2000029990) was conducted by Leng and colleagues to evaluate the injection of human BM-MSCs to 7 patients with Covid-19 pneumonia. BM-MSCs were administered intravenously at a dose of 1 million cells/kg PBW. After BM-MSC administration, patients were followed for 14 days to assess the safety and efficacy of BM-MSC treatment. Clinical benefits were observed in these patients, evidenced by pulmonary function improvement. The overactivated immune cells disappeared in 3–6 days, including CXCR3^+^CD4^+^ T cells, CXCR3^+^CD8^+^ T cells and CXCR3^+^ NK cells. Meanwhile, serum TNF-α levels were significantly decreased, and anti-inflammatory IL-10 levels were increased in BM-MSCs treatment group. Thus, human BM-MSCs-therapy may represent a safe and effective method for patients with Covid-19 pneumonia [91].

Until Mar. 10th 2020, guidelines to standardize stem cell treatment for Covid-19 were issued in China. The general protocol for MSC clinical application is that patients receive no more than 3 stem cell infusions, each infusion dose is 1–5 × 10^6^ cells/kg body weight, and each interval between infusions is no less than 3 days. Recently, a phase 1 Covid-19 clinical trial (parallel assigned controlled, non-randomized, n = 9 for each group) was reported to evaluate the safety of human UC-MSCs in patients with moderate and severe Covid-19 symptoms. The cells were infused three times on day 0, 3, and 6 at a dose of 3 × 10^7^ cells/infusion. All patients in this phase 1 trial recovered and were discharged, showing the safety of MSC intravenous infusion [92]. In a phase 2 Covid-19 clinical trial (randomized, double-blind, placebo-controlled, n = 65 for UC-MSCs group and n = 35 for placebo group), three cycles of UC-MSCs (4 × 10^7^ cells per infusion, on day 0, 3, and 6) or placebo were administrated to treat severe Covid-19 patients with lung damage. The proportions of whole lung lesion volumes were monitored from baseline to day 28. Compared to placebo, UC-MSCs infusion improved pulmonary function significantly, evidenced by reduced solid component lesion proportion [93]. These results primarily proved the therapeutic efficacy and safety of UC-MSCs in Covid-19 patients.

Importantly, human BM-MSCs were negative for ACE2 and TMPRSS2 genes, which indicated human BM-MSCs may be free from SARS-CoV-2 infection [91]. Moreover, a recent study reported that a Covid-19 patient was cured successfully with the intravenous infusion of human UC-MSCs and convalescent plasma. This combination therapy may have synergistic effects in inhibiting cytokine storm and improving pulmonary function [94].

ARDS in Covid-19 patients is often associated with the cytokine storm which causes host immune disorders. Neutrophils are critical mediators of severe SARS-CoV-2 infection, and contribute to organ damage and mortality in Covid-19 patients [95, 96]. Dysregulation of dendritic cells, Th17 cells and Treg cells have been observed in Covid-19 patients [97–99]. The activation and infiltration of inflammatory and immune cells can trigger an overproduction of cytokines, releasing multiple inflammatory mediators, such as IL-6, IFN-γ, and TNF-α [100, 101]. Therefore, the immunomodulatory properties of MSCs may be the most important aspect to benefit ARDS patients. In the UC-MSC or BM-MSC treatment patients, numerous cytokines showed the reduced trends, and the overactivated immune cells were decreased [91, 92]. Particularly, IL-6 was dramatically decreased in the patients with high baseline IL-6 levels after UC-MSC infusion for 3 days, but not in the patients with low IL-6 levels [92]. Therefore, the patient with high plasma cytokine concentration may benefit more from UC-MSCs treatment.

**Fig. 3:**
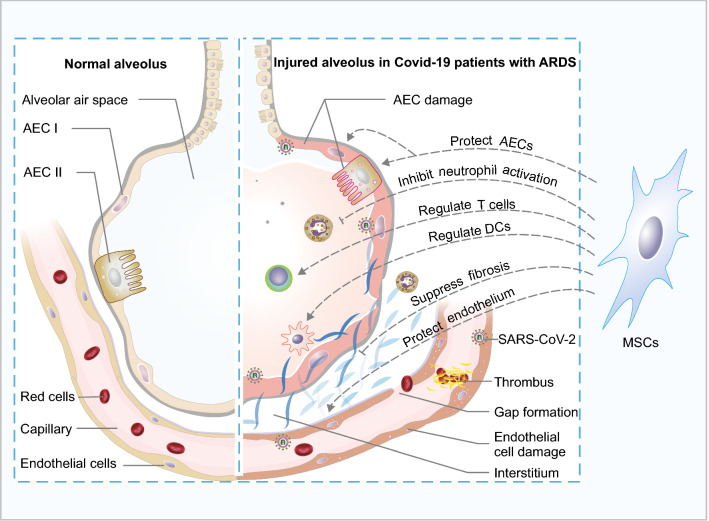
Lung pathophysiological changes in Covid-19 patients with ARDS were matched with MSC functions. The Covid-19 patients with ARDS may present with several clinical symptoms, including respiratory failure and disrupted endothelial cell membranes. Neutrophils and T cells are recruited to the injured endothelium, escape the capillary and pass through the lung interstitium into the alveolar air space, which is filled with edema fluid. Widespread vascular thrombosis and pulmonary fibrosis are also present in the injured capillary. *AEC I* type I alveolar epithelial cells, *AEC II* type II alveolar epithelial cells

Above all, since the therapeutic mechanisms of MSCs against ARDS were matched with the pathological characters of Covid-19, MSCs infusion can be considered as a cell-based therapy for Covid-19 patients (Fig. 3). Meanwhile, the long-term effects of MSCs on pulmonary function should be monitored in the following clinical trials.

## Problems and prospects

Although MSC-based therapy brings new hope for the treatment of ARDS, many challenges remain to be addressed before such therapy is routinely used in clinical applications. Infusion of MSCs via intravenous showed significant improvement of pulmonary function, while this route may cause dose-dependent pulmonary emboli or infarctions. Thus, the dose of cells should be strictly controlled. The intraperitoneal and intratracheal routes are rarely used in clinical trials despite the proven efficacy in pre-clinical studies [102, 103]. Extra medium inhalation during intratracheal MSC infusion may worsen ARDS, which should be noticed. Theoretically, intratracheal infusion of MSCs may benefit the AECs mostly, while intravenous injection may be in favor of the endothelial cells firstly. However, the optimal route of MSC administration for ARDS is probably intravenous infusion. Through this route, MSCs may also interact with the blood immune cells directly to inhibit cytokine storm. In pre-clinical experiments and clinical trials, the effects of different delivery routes should be compared to determine the superior injection route [81, 82, 104, 105].

The efficacy of MSCs infusion was not as promising as expected in some clinical trials. Future studies should focus on improving the efficacy of MSCs. It is reported that modulation of autophagy reveals the potential to increase the therapeutic efficacy of MSCs by enhancing their immunoregulatory abilities [106]. Infusion of rat BM-MSCs cultured under hypoxic conditions could promote cell survival and therapeutic efficacy [107]. Furthermore, the genetically modified rat and mouse BM-MSCs have enhanced beneficial effects to ameliorate lung tissue damage [62, 108, 109]. Therefore, precondition of MSCs is a promising strategy to improve the therapeutic ability of MSCs for ARDS [110].

After intravenous administration, MSCs can stay in the body for hours to days and gradually disappear. Thus, there may be no intermediate and long-term tumor risk. However, human and rat BM-MSC infusion may also promote tumor growth and angiogenesis by entering into the tumor microenvironment [111–113]. Therefore, the indications and contraindications of MSC infusion have to be clarified in future studies, especially in cancer patients. In 2019, an updated systematic review reported the adverse effects of MSCs after intravascular administration in 2696 patients. Compared to controls, MSC infusion was associated with an increased risk of transient fever [114]. Consistently, in the recent Covid-19 phase 1 trial, two patients (n = 9) developed transient facial flushing and fever after receiving UC-MSCs [92]. However, MSC infusion did not increase the risks of acute infusional toxicity, infection, pulmonary embolism, death or malignancy [114]. It's worth noting that lethal pulmonary thromboembolism after administration of AD-MSCs was indeed observed in clinical trials, and dose-dependent pulmonary embolism was also confirmed in mice after intravenous AD-MSC infusion [115]. Therefore, the dose of MSCs should be strictly controlled in clinical trials, and the cells should be dispersed into single cells before intravenous infusion.

In addition, MSCs hold the advantage of being manufactured as ready-to-use therapeutic products, because they can be used for allogeneic transplantation. However, standard procedures should be established to ensure the safety and efficacy of MSCs. During MSCs isolation and culture, the animal serum is a major concern and may cause undesirable complications. Instead, human AB serum (HABS) and human platelet lysate (HPL) were used for most xeno-free cultures. However, because of the donor heterogeneity, the quality of HABS and HPL may vary between batches. Another alternative is chemically defined media, which may enable stable MSC culture for clinic use [110, 116]. Importantly, precondition of MSCs represents a promising strategy to prime the cells with improved efficacy for specific diseases, including hypoxia, extracellular matrix, hormones, growth factors and so on [110]. These conditions increased the complexity of standard MSC production. Disease-specific MSC products should be standardized in the future. Recently, the human mesenchymal stem cell standard (T/CSCB 0003–2021) was issued in China, which may help standardized the production and application of MSCs.

## Conclusions

There are several potential mechanisms of MSC-based treatment in ARDS, including regulation of immune and inflammatory cells, paracrine of cytokines, the release of exosomes with benefits, modulation of endoplasmic reticulum stress and attenuation of pulmonary fibrosis. These properties enable MSCs to ameliorate ARDS. In pre-clinical studies, the infusion of MSCs clarified the therapeutic effects in ARDS models, and the results from Covid-19 clinical trials demonstrated the safety and potential efficacy of MSCs. However, the efficacy of MSC treatment should be confirmed further in larger trials, especially in Covid-19 patients with ARDS. Besides, studies are needed to define the optimal cell source, dose and route of MSCs therapies, and to provide an effective and safe treatment option for patients who suffer from ARDS, especially for Covid-19 patients.

## Data Availability

Not applicable.

## Abbreviations

AEC I: Type I alveolar epithelial cells
AEC II: Type II alveolar epithelial cells
ARDS: Acute respiratory distress syndrome
Covid-19: Coronavirus disease 2019
DCregs: Regulatory dendritic cells
ERS: Endoplasmic reticulum stress
iNOS: Inducible nitric oxide synthase
KGF: Keratinocyte growth factor
LPS: Lipopolysaccharide
mDCs: Mature dendritic cells
MSC: Mesenchymal stem cell
NETs: Neutrophil extracellular traps
NF-κB: Nuclear factor kappa-B
PBW: Predicted body weight
ROS: Reactive oxygen species
SARS-CoV-2: Severe acute respiratory syndrome coronavirus 2
Th17: T helper 17 cells
Treg cells: Regulatory T cells
